# Evaluation of peri-implant perfusion in patients who underwent avascular augmentation or microvascular reconstruction using laser Doppler flowmetry and tissue spectrophotometry: a prospective comparative clinical study

**DOI:** 10.1007/s00784-024-05825-w

**Published:** 2024-07-17

**Authors:** Marie Sophie Katz, Mark Ooms, Philipp Winnand, Marius Heitzer, Florian Peters, Kristian Kniha, Frank Hölzle, Ali Modabber

**Affiliations:** Department of Oral and Maxillofacial Surgery, University Hospital RWTH Aachen, Pauwelstraße 30, 52074 Aachen, Germany

**Keywords:** Laser-doppler flowmetry, Tissue spectrophotometry, Avascular graft, Microvascular bone graft, Perfusion, Hyperemia

## Abstract

**Objectives:**

The aim of this study was to evaluate the peri-implant perfusion, such as oxygen saturation, the relative amount of hemoglobin, and blood flow, in implants placed in pristine bone and avascular and microvascular grafts using a non-invasive measurement method.

**Materials and methods:**

A total of 58 patients with 241 implants were included. Among them, 106 implants were based in native bone (group I), 75 implants were inserted into avascular bone grafts (group II), and 60 implants were placed in microvascular bone grafts (group III). Gingival perfusion was measured using laser Doppler flowmetry and tissue spectrophotometry (LDF-TS). Implants with signs of gingival inflammation were excluded to analyze healthy implant perfusion in different bony envelopes.

**Results:**

The mean values for oxygen saturation, relative hemoglobin levels, and blood flow did not differ significantly between the groups (*p* = 0.404, *p* = 0.081, and *p* = 0.291, respectively). There was no significant difference in perfusion between implants that were surrounded by mucosa and implants based within cutaneous transplants (*p* = 0.456; *p* = 0.628, and *p* = 0.091, respectively).

**Conclusion:**

No differences in perfusion were found between implants inserted into native bone and implants involving bone or soft tissue augmentation. However, implants based in avascular and microvascular transplants showed higher rates of peri-implant inflammation.

**Clinical relevance:**

Peri-implant perfusion seems to be comparable for all implants after they heal, irrespective of their bony surroundings. Although perfusion does not differ significantly, other factors may make implants in avascular and microvascular transplants vulnerable to peri-implant inflammation.

## Objectives

Healthy peri-implant tissue and its physiological perfusion are fundamental requirements for a well-working defense against bacteria and a stable marginal bone level, which is crucial for the long-term survival of an implant [1–3]. Vascularization occurs to a lesser extent in peri-implant mucosa than in natural teeth because the implant tends to have a greater amount of collagen fibers attached to it than Sharpey’s fibers, and it resembles scar tissue [1]. The lack of natural periodontium around an implant causes peri-implant inflammation to progress faster than gingivitis, affecting natural teeth [4]. Berglundh et al. conducted an animal study and showed that while gingiva around natural teeth is vascularized by supraperiosteal vessels lateral to the alveolar process and vessels of the periodontal ligament, peri-implant mucosa is only perfused by terminal branches of larger vessels originating from the periosteum of the bone in the implant site [5]. This indicates that the quality of the bone in which the implant is positioned is crucial for peri-implant mucosa perfusion. Bony atrophy or larger defects sometimes lead to the need for augmentation of avascular or microvascular bone grafts for rehabilitation with dental implants to be possible. Graft type and size are dependent on the location and extent of the defect and the mobilization of intraoral tissues, which leads to scars in the underlying connective tissue and alterations in the periosteum [6–11].

The revascularization and perfusion of a graft depends on the density of the bone and its vascular nutrition, which is important for bone level stability and to avoid ongoing resorption processes [12, 13]. As the aim of dental reconstruction is to remain as close as possible to the original dental situation, it is important to note that implants in avascular and microvascular transplants seem to have a greater risk of peri-implant inflammation and lower survival rates overall [14, 15]. The quality of the peri-implant bone and mucosa plays a role in the onset of peri-implant mucositis, and patient-related factors and habits also contribute to a pathological environment around the implant [16–19].

To understand and detect changes in gingival micro perfusion, attempts have been made to visualize gingival blood flow using non-invasive measurements such as ultrasonography [20, 21] and laser Doppler flowmetry and tissue spectrophotometry (LDF-TS) [22–24]. LDF-TS is a frequently used tool in maxillofacial and plastic surgery to monitor microvascular transplants extra- and intraorally [25–29], as it can help identify oxygen saturation (SO_2_; in %), the relative amount of hemoglobin (rHb; in arbitrary units [AU]), and blood flow (in AU). A previous study showed that these perfusion parameters significantly differ among patients with gingival inflammation at the papilla [22].

The primary aim of this study was to evaluate peri-implant soft tissue perfusion. The secondary aim was to analyze whether LDF-TS measurements correlate with different bony surroundings, the presence of keratinized tissue or a cutaneous transplant, gingival biotype, smoking habits, or a history of radiotherapy.

The aim of this study was to evaluate the peri-implant perfusion, such as oxygen saturation, the relative amount of hemoglobin, and blood flow, in implants placed in native bone and avascular and microvascular grafts using a non-invasive measurement method.

## Materials and methods

### Study design

This study was approved by the local clinical research ethics committee (Decision Number 23–097) and registered at the German Clinical Trials Register (File Number DRKS00032148). All procedures performed in this study were in accordance with the ethical standards of the 1964 Helsinki Declaration. Informed consent was obtained from all participants included in the study.

### Eligibility criteria

All participants were recruited during their regular implant recalls at the Department of Oral and Maxillofacial Surgery at the University Hospital RWTH Aachen, Germany. To be included in this study, patients had to be at least 18 years of age and have at least one dental implant inserted into native bone, an avascular bone graft, or a microvascular bone transplant. The patients who underwent an avascular augmentation before implantation had a bony atrophy or defect without any discontinuity of the maxilla or mandible. The patients who had a reconstruction with a microvascular transplant (i.e. fibula flap or a microvascular iliac crest transplant) had a larger defect due to oncologic, traumatic or cystic processes before which could only be restored by a transplant with vascularization.

Patients with acute intraoral swelling, those who had smoked less than two hours before their examination, and pregnant patients were excluded. In addition, implants with any signs of gingival inflammation were excluded to ensure a healthy study population for detecting the baseline perfusion characteristics of each graft.

Based on the reviews conducted by Heitz-Mayfield et al. [30] and Renvert et al. [31], peri-implant mucositis was defined as the presence of bleeding on probing (BOP) at the papilla, with concomitant clinical signs of inflammation, such as redness, swelling, or suppuration. Implants with BOP, clinical signs of inflammation, and probing depths ≥ 6 mm were considered affected by peri-implantitis [31, 32].

### Sample size calculation

The existing literature on clinical trials of gingival and peri-implant perfusion methods was reviewed to calculate a suitable range for the sample size. In particular, the required sample size was derived based on a study by Barootchi et al. [20], who used color flow ultrasonography to evaluate peri-implant tissue perfusion in 21 patients with 42 dental implants, as well as our former study on gingival perfusion in patients with and without gingivitis [22]. Since the intraoral use of LDF-TS is relatively new, the basal flow differences were the primary outcomes considered when calculating the sample size.

The statistical program G* Power Version 3.1.9.6 (Heinrich-Heine-Universität, Düsseldorf, Germany) was used for the calculation, with an alpha value of 0.05, an effect size of 0.75, and a statistical power of 90%. Based on these parameters, a sample size of at least 45 patients was found to reject the null hypothesis that there is no significant difference concerning primary basal flow differences, with 90% power and a 95% confidence interval.

### Former bone augmentation procedures

The patients who underwent bone augmentation procedures prior to implantation were all augmented with only autologous bone transplants. The avascular transplants, such as retromolar, avascular iliac crest or calvaria bone had a healing period of 3–6 months before implantation. The microvascular transplants, such as fibula free flaps and microvascular iliac crest transplant were engrafted for bony healing over 9–12 months before dental implants were placed. All implants were prosthetically restored after a healing period of at least three months.

### Perfusion measurement

**Fig. 1:**
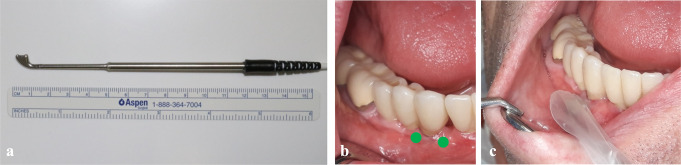
The gingival probe of the “oxygen to see” (O2C) device (**a**), the mesial and distal measurement points on the buccal side of the implant (**b**) and the intraoral measurement (**c**)

All examinations started with perfusion measurements via LDF-TS to avoid manipulating the peri-implant tissues by probing. The “oxygen to see” (O2C) device (LEA-Medizintechnik, Gießen, Germany) is frequently used to monitor microvascular transplants in maxillofacial and plastic surgery. It measures SO_2_ (%), rHb (AU), and blood flow (AU) with a laser and a white light. Its intraoral use is a relatively new development, as it requires a specialized small probe, such as the LSX-41 gingival probe (LEA-Medizintechnik, Gießen, Germany), which was used in our study (Fig. 1).

The LSX-41 gingival probe has a dimension of 5 × 2 mm and a measurement depth of 1 mm. It is specifically manufactured for intraoral measurements and consists of two small batches next to the sensor and the laser that ensure its stable placement on adjacent teeth, without any noteworthy compression of the tissue measured.

Prior to perfusion measurement, the patient was laid in a horizontal position, and the light on the dental chair was switched off to avoid interference during the measurement. Each implant was measured mesially and distally on the buccal side to determine the mean value of peri-implant perfusion (Fig. 1). Each measurement was recorded for 10 s, prior to which the examiner had five seconds to adjust the probe head and to make sure that there were no micromovements during the recording period. If there were any signs of unexpected movement or disturbance, each measurement had to be immediately repeated.

### Clinical classification

After completing the perfusion measurements, conventional clinical examinations of the implants were conducted to determine the gingival biotype, presence of keratinized gingiva or cutaneous transplants (i.e. radial forearm flaps, anterolateral thigh flaps, free fibula flaps or split skin grafts) next to the implant, and signs of clinical inflammation. Implants with peri-implant mucositis or peri-implantitis were excluded from our analysis so that the baseline values for healthy implant perfusion could be determined. The gingiva type was considered thin if the periodontal probe was visible through the sulcus, if there was no transparency, the biotype was considered thick [33, 34]. The results of the perfusion measurements and clinical examinations were subsequently analyzed, and correlations were evaluated.

### Statistical analysis

For the categorical data (sex, smoking habits, implant location, biotype, presence of keratinized gingiva and a cutaneous transplant, and presence of peri-implant inflammation), the differences between the groups (I: native bone; II: avascular bone grafts; III: microvascular bone transplants) were analyzed using a chi-square test or the Freeman–Halton test. Metric data (age, mean SO_2_, mean rHb, and mean blood flow) were evaluated using the Kruskal–Wallis test, as they lacked a Gaussian distribution according to the Shapiro–Wilk test. P-values < 0.05 were considered significant.

## Results

A total of 60 patients (32 male, 28 female) with 273 implants were recruited. Among them, 32 implants with peri-implant inflammation, such as peri-implant mucositis or peri-implantitis, were excluded. Of the implants excluded, 16 were positioned in microvascular bone transplants, 14 were in avascular bone grafts, and two were in native bone. Our final study sample included 58 patients with 241 implants, of which 106 implants were placed in native bone, 75 implants were positioned in avascular bone grafts (51 in avascular iliac crest grafts, 11 in retromolar bone grafts, and 13 in calvaria split bone), and 60 implants were positioned in microvascular bone grafts (45 in fibula grafts and 15 in microvascular iliac crest grafts).

There were significant differences in the prevalence of peri-implant inflammation between the groups: 21.05% of the implants placed in microvascular transplants, 15.73% of the implants in avascular bone grafts, and only 1.85% of the implants in native bone were affected by peri-implant mucositis or peri-implantitis (*p* < 0.001). The mean age of the patients with implants in native bone, patients with avascular transplants, and patients who had undergone microvascular reconstruction were 66.6 years (SD ± 10.8), 62.2 years (SD ± 10.1), and 55 years (SD ± 14.4), respectively. There were no statistical differences in the sex (*p* = 0.979) of the patients, but the age distributions (*p* = 0.040) of the three groups showed that patients with avascular and microvascular transplants were, on average, significantly younger.

The study sample included five patients with regular smoking habits, of whom four belonged to the group with implants in native bone, and one had a microvascular bone transplant. Nevertheless, all patients confirmed that they had not smoked for at least two hours before the measurements were taken. Smoking habits did not significantly differ between the groups (*p* = 0.116).

Patients who had undergone radiotherapy accounted for 14 implants; all of these implants were placed in microvascular transplants that had not been radiated themselves. Further, 188 implants were of a thick gingival biotype (66 in native bone, 68 in avascular grafts, 54 in microvascular grafts), and 53 had a thin biotype (40 in native bone, 7 in avascular grafts, and 6 in microvascular grafts). The gingiva-type distributions of the groups significantly differed in favor of the thick biotype (*p* < 0.001). The presence of keratinized gingiva (*p* < 0.001) and the distribution of cutaneous transplants located next to the implants (*p* < 0.001) also differed between the groups (Table 1).

**Table 1:** Patient collective and group-based characteristics of the implants measured

	Native bone (Group I)	Avascular graft (Group II)	Vascular graft (Group III)	Total	*p*-value
Sex					
Male	11 (50%)	10 (52.6%)	9 (52.9%)	30 (51.7%)	0.979
Female	11 (50%)	9 (47.4%)	8 (47.1%)	28 (48.3%)	
**Mean age**					
	66.6 (SD ± 10.8)	62.2 (SD ± 10.1)	55.0 (SD ± 14.4)	61.7 (SD ± 12.5)	0.040*
**Smoking habits**					
Yes	4 (18.2%)	0 (0%)	1 (5.9%)	5 (8.6%)	0.116
No	18 (81.8%) (%)	19 (100%)	16 (94.1%)	53 (91.4%)	
**Radiation**					
Yes	0 (0%)	0 (0%)	5 (29.4%)	5 (8.6%)	< 0.001*
No	22 (100%)	19 (100%)	12 (70.6%)	53 (91.4%)	
**Gingival biotype**					
thick	66 (62.3%)	68 (90.7%)	54 (90%)	188 (78%)	< 0.001*
thin	40 (37.7%)	7 (9.3%)	6 (10%)	53 (22%)	
**Keratinized gingiva**					
Yes	58 (54.7%)	12 (16%)	20 (33.3%)	90 (62.7%)	< 0.001*
No	48 (45.3%)	63 (84%)	40 (66.7%)	151 (37.3%)	
**Cutaneous transplant next to the implant**					
Yes	7 (6.6%)	8 (10.7%)	32 (53.3%)	47 (19.5%)	< 0.001*
No	99 (93.4%)	67 (89.3%)	28 (46.7%)	194 (80.5%)	

*Legend* For the categorical data (sex, smoking habits, implant location, radiation, biotype, presence of keratinized gingiva, and cutaneous transplant), the differences between groups were analyzed using chi-square tests or the Freeman–Halton test. The metric data (age, mean probing depth, mean oxygen saturation, mean rHb, and mean blood flow) were evaluated using the Kruskal–Wallis test, as they lacked a Gaussian distribution according to the Shapiro–Wilk test. P-values < 0.05 were considered significant*

**Fig. 2:**
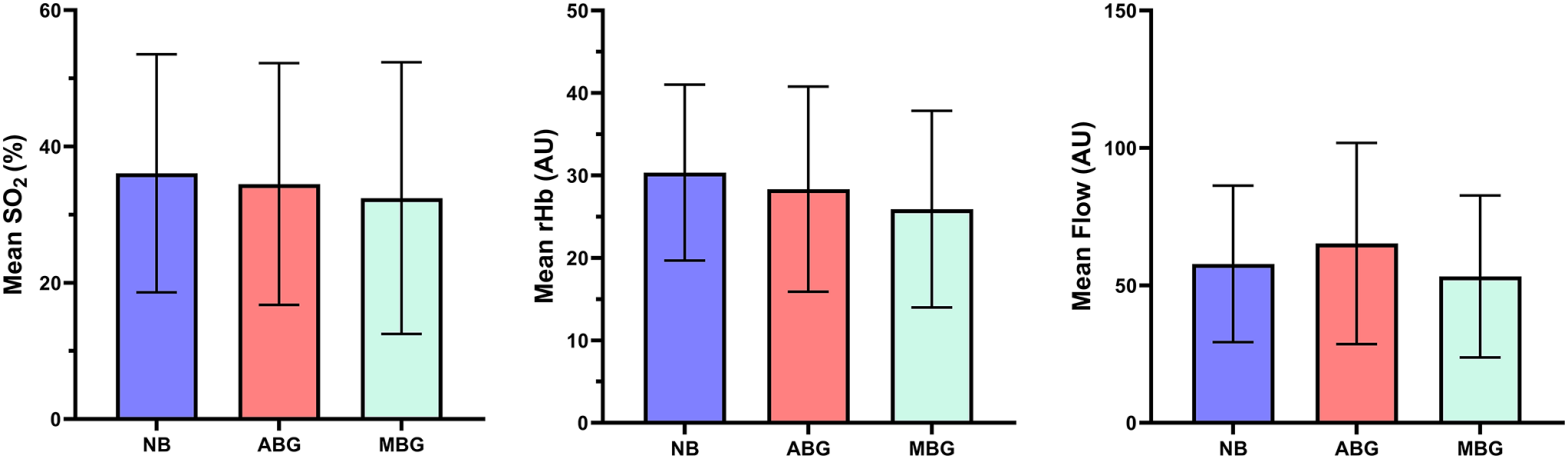
Comparison of perfusion values for implants placed in native bone (NB), and in avascular (ABG) and microvascular bone grafts (MBG)

A comparison of the 194 healthy implants that were surrounded by mucosa showed that the mean SO_2_ (%), rHb (AU), and blood flow (AU) values did not differ significantly between implants placed in native bone, avascular grafts, or microvascular bone grafts surrounded by mucosa (*p* = 0.404, *p* = 0.081, and *p* = 0.291, respectively) (Fig. 2).

The bars represent the mean values (SO_2_, rHb, and flow), and the whiskers indicate the SDs.

**Fig. 3:**
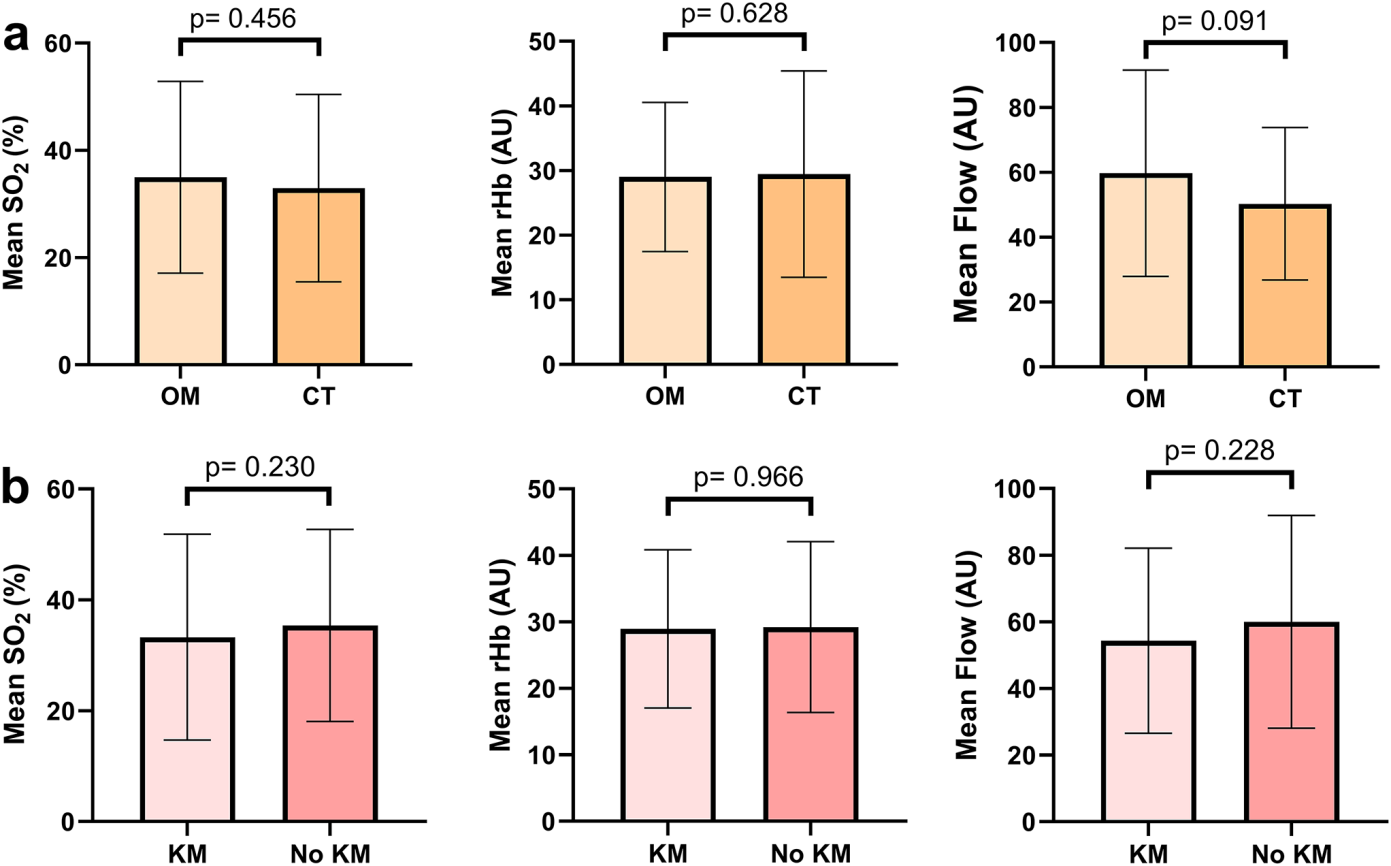
Comparison of perfusion values for (**a**) implants surrounded by oral mucosa (OM) and cutaneous transplants (CT), and (**b**) for implants with and without keratinized mucosa (KM)

Comparing the peri-implant perfusion of implants surrounded by mucosa and implants next to a cutaneous transplant revealed no significant differences in the mean SO_2_ (%), rHb (AU), and blood flow (AU) values (*p* = 0.456; *p* = 0.628, and *p* = 0.091, respectively; Fig. 3). No significant differences were seen in the mean SO2, rHb, and blood flow values for peri-implant perfusion with and without the presence of keratinized peri-implant tissue (*p* = 0.230, *p* = 0.966, and *p* = 0.228, respectively; Fig. 3).

The bars represent the mean values (SO_2_, rHb, and flow), and the whiskers indicate the SDs.

The mean SO_**2**_(*p* = 0.112), rHb (*p* = 0.481), and blood flow (*p* = 0.516) values did not differ between peri-implant tissues that had undergone radiotherapy and nonradiated areas.

Smoking also had no significant impact on the perfusion parameters measured (mean SO_2_: *p* = 0.588; mean rHb: *p* = 0.155; mean flow: *p* = 0.935).

## Discussion

A few former studies have evaluated peri-implant vascularization after different oral procedures [20, 21, 35], but the role and influencing factors of superficial peri-implant soft tissue perfusion remain mostly unclear. While gingiva around natural teeth substantially differs from that around implants, peri-implant tissue can also be quite heterogeneous because some implants require prior hard or soft tissue augmentation [36, 37].

LDF-TS is a common tool used to monitor microvascular transplants [25–29] and has been validated for intraoral mucosa measurements in former studies [22, 23]. To use LDF-TS for regular implant recalls, the mean parameters for physiological peri-implant perfusion have to be established, and potential differences in peri-implant perfusion due to former augmentations must be addressed. The present study was the first to evaluate peri-implant perfusion and the impact of former bone transplantation on soft tissue perfusion.

In this study, peri-implant perfusion parameters measured by LDF-TS did not significantly differ between patients with implants placed in native bone and those with implants following an avascular or microvascular augmentation procedure. This suggests that although extensive operations such as microvascular augmentation led to tissue alteration and scarring, a directly adjacent peri-implant perfusion remains unaffected by these procedures or is almost fully regenerated after soft tissue healing. This is in line with Griffin et al.’s reports of minimal scarring of the oral mucosa, which was associated with delayed inflammation and a muted angiogenic response [38].

Milstein et al. [24], who compared micro perfusion in normal and alveolar cleft gingiva, found significant differences in blood flow and capillary density but no differences in oxygen saturation and relative hemoglobin values. This could have been due to the high amount of scarring of the soft tissue after multiple cleft operations compared to a single augmentation procedure, which does not seem to cause extensive fibrosis in the alveolar gingiva. Upon comparing the micro perfusion of oral mucosa next to implants with adjacent cutaneous grafts, no significant differences were found in the mean oxygen saturation, relative hemoglobin, and blood flow values. It is known that cutaneous transplants show signs of metaplasia when healing next to the oral mucosa [39], which could be an explanation for the similar superficial peri-implant vascularization.

An adequate width of keratinized gingiva is known to be an important factor for maintaining peri-implant health [36]. However, peri-implant micro perfusion did not differ significantly between patients with and without keratinized gingiva in our study. This implies that it is less a matter of vascularization and more to do with the stability of keratinized gingiva and a small marginal gap that protects the implant from pathological influences. In our study population, patients who had undergone an avascular or vascular augmentation had significantly less keratinized tissue around their implants than patients who had implants based in native bone, which made them more vulnerable to peri-implant diseases [40].

Although it is known that smoking reduces the vascularization of peripheral vessels, no significant difference was found between smokers and non-smokers in this study [41]. This is in line with the findings of a former study [22], wherein gingival perfusion around natural teeth was evaluated. However, Rifai et al. found that smoking resulted in a redistribution of small and large vessels in the superficial and deeper connective tissue areas of the gingival papilla compared to nonsmoking patients [42]. Furthermore, the degree of vessel constriction and damage is associated with the number and the history of smoking, so this result might not be clearly seen in such a small population of smokers [43]. Nevertheless, it should be noted that smoking was not permitted for at least two hours prior to the clinical examinations, hence the effect of smoking might be more present directly after smoking. As this study mainly focused on perfusion of implants surrounded by different soft and hard tissue, this is a limitation which should be addressed in future studies comparing peri-implant perfusion in smokers to non-smokers.

Patients with microvascular bone transplantations are likely to also be affected by former radiation. In our study population, radiation did not affect the micro perfusion of the soft tissue around the implants. This is consistent with the findings of a study by Helmers et al., who evaluated oral micro perfusion with a CytoCam video microscope system and found no significant differences in gingival microcirculatory blood flow between patients with and without former radiation therapy [44]. A history of radiation remains a risk factor for implant survival, as xerostomia affects the immune response to peri-implant infections [30]. However, it must be stated, that in this study only 5 patients with 25 implants were had a history of radiation in the head and neck area, which is a small sample size. Further research comparing the perfusion of healthy gingiva to radiated mucosa is needed to fully understand the degree of vessel degeneration and its impact on the micro perfusion of the papilla.

When comparing the healthy perfusion at the papilla of natural teeth from the mentioned former study [22] to the perfusion values from this study, it is found that the mean SO_2_ was higher (healthy teeth: 33.1% vs. healthy implants: 34.6%), mean rHb was lower (healthy teeth: 33.8 AU vs. healthy implants: 29.1 AU), and mean blood flow was higher (healthy teeth: 42 AU vs. healthy implants: 58 AU) in the latter. This indicates that peri-implant tissues have lower oxygen intake levels, with the tissue quality favoring vessel congestion, and higher blood flow compensation. These features may account for a less capable immune defense against outer influences and high vulnerability compared to natural teeth.

In this study, a gingival probe that measured perfusion at a depth of 1 mm was used. As microcirculation of the gingival papilla is not fully understood yet, there might be vascular alterations to compensate hypoxia and vessel degenerations, which were not assessed with this probe.

Bone transplants tend to exhibit different resorption patterns and vascularization abilities due to their densities [10, 12, 14], but this does not seem to affect superficial perfusion directly next to an implant as long as there are no signs of clinical inflammation. Nevertheless, implants in avascular and microvascular transplants had a high share of peri-mucositis and peri-implantitis. This finding is in line with two retrospective studies by Blake et al. [14] and Wiesli et al. [15], which showed high rates of soft tissue inflammation and peri-implantitis and low implant survival rates in patients with microvascular transplants. The reasons for this vulnerability are probably multifactorial, and since peri-implant tissue perfusion seems to play an inferior role in this regard, the lack of keratinized gingiva may be one of the main factors affecting peri-implant health in augmented areas.

## Conclusion

LDF-TS is a rather new, non-invasive method that can contribute to understanding peri-implant perfusion and physiological vascularization around dental implants. It can be used to quantify mean oxygen saturation, relative hemoglobin levels, and blood flow and can thus provide insights into tissue condition and nutrition. There is no difference in peri-implant perfusion between implants inserted into native bone and implants following bone or soft tissue augmentation. However, implants based in avascular and microvascular transplants show higher rates of peri-implant inflammation, which may be associated with a lack of keratinized tissue.

### Clinical relevance

Peri-implant perfusion seems to be comparable for all implants after they heal, irrespective of their bony surroundings. Although micro perfusion does not differ significantly, other factors may make implants in avascular and microvascular transplants vulnerable to peri-implant inflammation.

## Data Availability

No datasets were generated or analysed during the current study.

## Abbreviations

LDF-TS: Laser Doppler Flowmetry and Tissue Spectrophotometry
O2C device: “Oxygen to see” device
SO_2_: Oxygen Saturation
rHb: relative amount Of Hemoglobin
AU: Arbitrary Unit
